# Population-genomic variation within RNA viruses of the Western honey bee, *Apis mellifera*, inferred from deep sequencing

**DOI:** 10.1186/1471-2164-14-154

**Published:** 2013-03-07

**Authors:** Robert Scott Cornman, Humberto Boncristiani, Benjamin Dainat, Yanping Chen, Dennis vanEngelsdorp, Daniel Weaver, Jay D Evans

**Affiliations:** 1 Bee Research Laboratory, USDA-ARS, Beltsville, MD, 20705, USA; 2 Swiss Bee Research Centre, Agroscope-Liebefeld-Posieux Research Station, Bern, 3003, Switzerland; 3 Department of Entomology, University of Maryland, College Park, MD, 20742, USA; 4 Bee Power, LP, Navasota, TX, 77868, USA; 5 Current address: Leetown Science Center, USGS, Leetown, Kearneysville, WV, 23540, USA; 6 Current address: Department of Biology, University of North Carolina, Greensboro, NC, 27402, USA

**Keywords:** Apis mellifera, Deformed wing virus, Israel acute paralysis virus, Single nucleotide polymorphism, Population genomics

## Abstract

**Background:**

Deep sequencing of viruses isolated from infected hosts is an efficient way to measure population-genetic variation and can reveal patterns of dispersal and natural selection. In this study, we mined existing Illumina sequence reads to investigate single-nucleotide polymorphisms (SNPs) within two RNA viruses of the Western honey bee (*Apis mellifera*), deformed wing virus (DWV) and Israel acute paralysis virus (IAPV). All viral RNA was extracted from North American samples of honey bees or, in one case, the ectoparasitic mite *Varroa destructor*.

**Results:**

Coverage depth was generally lower for IAPV than DWV, and marked gaps in coverage occurred in several narrow regions (< 50 bp) of IAPV. These coverage gaps occurred across sequencing runs and were virtually unchanged when reads were re-mapped with greater permissiveness (up to 8% divergence), suggesting a recurrent sequencing artifact rather than strain divergence. Consensus sequences of DWV for each sample showed little phylogenetic divergence, low nucleotide diversity, and strongly negative values of Fu and Li’s D statistic, suggesting a recent population bottleneck and/or purifying selection. The Kakugo strain of DWV fell outside of all other DWV sequences at 100% bootstrap support. IAPV consensus sequences supported the existence of multiple clades as had been previously reported, and Fu and Li’s D was closer to neutral expectation overall, although a sliding-window analysis identified a significantly positive D within the protease region, suggesting selection maintains diversity in that region. Within-sample mean diversity was comparable between the two viruses on average, although for both viruses there was substantial variation among samples in mean diversity at third codon positions and in the number of high-diversity sites. F_ST_ values were bimodal for DWV, likely reflecting neutral divergence in two low-diversity populations, whereas IAPV had several sites that were strong outliers with very low F_ST_.

**Conclusions:**

This initial survey of genetic variation within honey bee RNA viruses suggests future directions for studies examining the underlying causes of population-genetic structure in these economically important pathogens.

## Background

In addition to producing honey and other hive products, managed honey bee colonies are vital for the pollination of many crops. Pests and diseases that impact honey bees are therefore of economic concern, particularly in light of the long-distance transport of bees, bee products, and beekeeping equipment that can facilitate their spread. High rates of unexplained colony loss in recent years [1,2] have heightened these concerns and provided impetus for surveys of geographic and genetic variation in honey bee pathogens [3,4].

A number of RNA viruses have been recognized as major disease agents of honey bees. In addition to the recognized morbidity and mortality that can result from overt viral infections, lower viral loads that do not produce visible symptoms might nonetheless impact colony health as well. For example, viral loads are negatively associated with colony survival [5,6] and there is correlational evidence of synergism among viruses in Colony Collapse Disorder (CCD) [4]. Interactions have also been identified between viruses and other parasites, particularly the ectoparasitic mite *Varroa destructor*[6-8]. Recent genomic studies have identified novel viruses that may also be relevant to colony health, but about which little is yet known [3,4]. Additionally, recombination between co-infecting RNA viruses may facilitate adaptation or the emergence of novel viruses [9,10]. It has therefore become a major goal to better understand the diversity of viral species in honey bee populations and how they may interact during co-infection.

Understanding the genetic diversity *within* a viral species is important as well. RNA viruses in particular have high mutation rates, can evolve rapidly, and may exhibit adaptation to specific hosts and/or tissues. Virulence can depend strongly on viral genotype (as evidenced by the effects of specific point mutations on the virulence of human influenza virus [11] and attenuated polioviruses [12]), although host genotype, infection mode, and environment are likely to be important as well. Furthermore, the design and interpretation of studies of host specificity, host adaptation, and virus dispersal depend on an adequate baseline of standing genetic variation and population structure. This is in part due to a reliance on PCR-based methods for high-throughput detection and quantification of viruses, the validity of which depends on an adequate understanding of sequence variation for primer design. More fundamentally, the components of population-genetic variance themselves can provide important biological insights on viral demography, mutation rate, patterns of selection, and modes of replication.

In this study, we mined existing Illumina sequencing data to evaluate genomic variation within two well-characterized and economically significant RNA viruses of honey bees, deformed wing virus (DWV) [13] and Israel acute paralysis virus (IAPV) [14]. Both viruses are in the picornavirus-like clade of single-strand RNA viruses. Each virus is part of a larger complex of related strains or species that also infect honey bees: DWV and Varroa destructor virus 1 (VDV-1) are in the Iflaviridae; IAPV, Acute bee paralysis virus (ABPV), and Kashmir bee virus (KBV) are in the Dicistroviridae. DWV is common in managed honey bee colonies, is associated with a crumpled wing phenotype in highly infected adults, and, in addition to horizontal and vertical transmission among bees, is vectored by Varroa mites [15-17]. The titer of the replicating (negative) strand of DWV in mites has been shown to be predictive of DWV-associated pathology in bees [16]. IAPV is the most recently described honey bee dicistrovirus and can be highly virulent [18]. IAPV was implicated in CCD in early work [19] but this association has not recurred in subsequent studies [4,20].

Our results buttress previous studies which found a phylogenetic distinctiveness of IAPV in North America relative to other continents [21] and a likely selective sweep underlying reduced genetic variation among DWV isolates [22]. In addition, sliding-window analyses of single nucleotide polymorphisms (SNPs) indicated that regions under distinct selection pressures are differentiable from the genome background. SNP-diversity spectra differed among samples and may reflect factors such as the infection titer and number of hosts in each sample. Collectively, the data support the use of deep sequencing to investigate population-genetic variation in these viruses, ultimately to correlate viral genotypes with infection outcome.

## Methods

### Samples

We analyzed seven Illumina cDNA sequencing runs, each a single lane on an Illumina GAII Genome Analyzer, with read lengths of 67–80 bp. Two samples, termed CCD- and CCD+, are described in [4]. Briefly, eight workers each were collected from 61 hives diagnosed with CCD and from 63 hives that were nominally healthy. These hives were from temporary commercial apiaries in the United States. Another sample (BP) derived from abdominal tissues (DV, unpublished data) with evident scarring, malformation, or discoloration of the sting gland, pyloric valve, or Malpighian tubules. The WEAV sample represents six worker bees collected from Bee Weaver Apiaries, Navasota, Texas. The LARV sample represents ten larvae from a research apiary at the University of Georgia. The BRL sample represents 15 pupae from the Beltsville Bee Research Lab apiary that had been inoculated with a stock solution containing high levels of IAPV and DWV (purified from infected bees by maceration, followed by 0.2μm filtration and centrifugation against a 20% sucrose cushion). The VARROA sample represents a pool of approximately 1,000 phoretic female mites that were collected from this same research apiary by dislodging them from adult worker bees in the hive. These samples, which were collected and sequenced for other research purposes, represent a diverse set of bee life-history stages and different numbers of contributing individuals.

### Sample preparation and sequencing

Details for sample RNA extraction varied among the different samples, but all used the Trizol reagent and manufacturer's recommended protocol. cDNA synthesis was performed with SuperScript II (Invitrogen) and primed with combinations of short random oligonucleotides and/or poly-dT primers. Methodological variation is a potentially important cause of variation in coverage across a viral genome in different samples, but ultimately is not germane to the goals of this study, which investigated polymorphism only at those sites with sufficient coverage irrespective of why coverage may have varied among sites or across samples.

### Sequence analysis

Sequencing accessions are aggregated under NCBI BioProject PRJNA172020, and individual accessions are given in Table 1. Reads were mapped with BWA [23] using a polymorphism limit of 4% and a seed length of 28 bp. Consensus sequences were generated from the resulting alignments by identifying the most frequent base ('plurality rule' [24]). If a position had zero coverage, the ambiguous character 'N' was used. Indels were not permitted. Consensus-sequence analysis of nucleotide diversity (π), the number of segregating sites (S), and Fu and Li’s D were calculated with windows of 200 bases and a 25-base step, using DnaSP [25]. Phylogenetic trees were created with MEGA5 [26] using the Tamura 3-parameter nucleotide model and the neighbor-joining algorithm; alternative methods produced concordant phylogenies. Bootstrap values of trees were based on 1,000 replicates.

**Table 1:** Number of reads mapped to viral reference genomes from each Illumina sequencing run

**Sample**	**Accession**	**Description**	**Total reads**	**DWV**	**VDV-1**	**IAPV**	**ABPV**	**KBV**
BP	SRX180863	Dissected Malpighian tubules, pyloric valve, and sting glands from 8–50 workers from multiple California colonies	21,057,716	349,567	38	7,600	0	24
CCD-	SRX028145	504 adult worker abdomens from 63 colonies across U.S.	22,103,561	899	0	3,818	0	0
CCD+	SRX028143	488 adult worker abdomens from 61 colonies across U.S.	53,040,678	8,165	1	773	13	93
BRL*	SRX210759	15 pupae from a single Maryland colony	29,089,788	384,052	3	15,548,490	0	0
LARV	SRX180864	10 4th-instar larvae from a single Georgia colony	30,295,220	149,139	0	2,028,649	0	0
WEAV	SRX201544	6 workers from a single Texas colony	27,877,305	648,871	1	0	0	0
VARROA	SRX174087	~1,000 mites pooled from several colonies at a single Maryland site	22,920,031	2,758,133	6	0	0	0

*DWV*= Deformed wing virus, *VDV-1* = Varroa destructor virus-1, *IAPV* = Israeli acute paralysis virus, *ABPV* = Acute bee paralysis virus, *KBV* = Kashmir bee virus.

*RNA from virus-enriched sample, see Materials and Methods.

SNP haplotype diversity was calculated as the gene diversity at each position having at least 10X coverage, using the formula (N/(N-1))*(1 – Σ(p_*i*_)^2^) where p_*i*_ is the frequency of each of the four nucleotides at that position and N is the total number of reads mapped at that position [27]. Haplotype diversity at a site depends only on the frequency of each base and is mathematically equivalent to haplotype diversity for single positions and to expected heterozygosity for diploid populations. SNP haplotype diversity was also calculated for sliding windows of 100 bases and a 25-base step. (Note that π cannot be computed directly from sequence reads because, by the nature of short-read sequencing, large contiguous sequences that align end-to-end are not available.) F_ST_ values were calculated for sliding windows of 100 bp with a step size of 25 bp, using popoolation2 [28]. A distribution of F_ST_ values for random 100-bp windows was estimated by randomly re-sampling sites to generate 100,000 artificial intervals for each virus.

## Results and discussion

### Samples and read mapping

The seven Illumina short-read data sets analyzed are listed in Table 1. Two runs (CCD+ and CCD-) were from a survey of worker bees from colonies diagnosed with CCD or nominally healthy controls, respectively. These samples were collected across a broad geographic range in the U.S. as part of targeted surveys for known bee pathogens [4]. One run (BP) combined three tissues (sting gland, pyloric valve, and Malpighian tubules) that exhibited gross pathologies or abnormalities after dissection from worker bees (DV, unpublished data). Another run (BRL) was of pupae intentionally inoculated with IAPV and DWV at the Beltsville Bee Research Laboratory. The remaining sequencing runs did not have overt disease or *a priori* expectation of viral infection: a) workers collected from Bee Weaver Apiaries, Navasota, Texas, U.S. (WEAV); b) larvae collected from a research apiary at the University of Georgia (LARV); and c) Varroa mites collected from the Beltsville Bee Research Laboratory.

Read mapping was initially done competitively (i.e., all references are considered simultaneously rather than one at a time) for DWV, IAPV, and their close relatives using parameters described in Materials and Methods. This was done to determine the predominant viral species present in each sample and to evaluate whether sequence reads originating from a related species might be attributed incorrectly to the species of interest. For DWV, reads were mapped to DWV accession NC_004830.2 and VDV-1 accession NC_006494.1. For IAPV, reads were mapped to IAPV accession NC_009025.1 initially, ABPV accession NC_002548.1, and KBV accession NC_004807.1.

The number of reads mapping to each accession for each sample is shown in Table 1. Although VDV-1 and recombinants between VDV-1 and DWV have been found in honey bee samples from Europe [29,30], we detected zero or a trivial number of reads for VDV-1 in all samples, including the Varroa sample. DWV was present in all seven samples, but with low coverage in the CCD- sample. Five samples had reads mapping to IAPV, one of which (CCD+) had appreciable numbers of reads mapping to the related virus KBV, but at a much lower frequency than IAPV (10.6% of all CCD+ reads mapping to these viruses). The presence of KBV reads could potentially bias SNP estimation for IAPV in this sample, but given that the two accessions listed above have a genome-average identity of only 77.6%, we believe this effect should be negligible.

**Figure 1:**
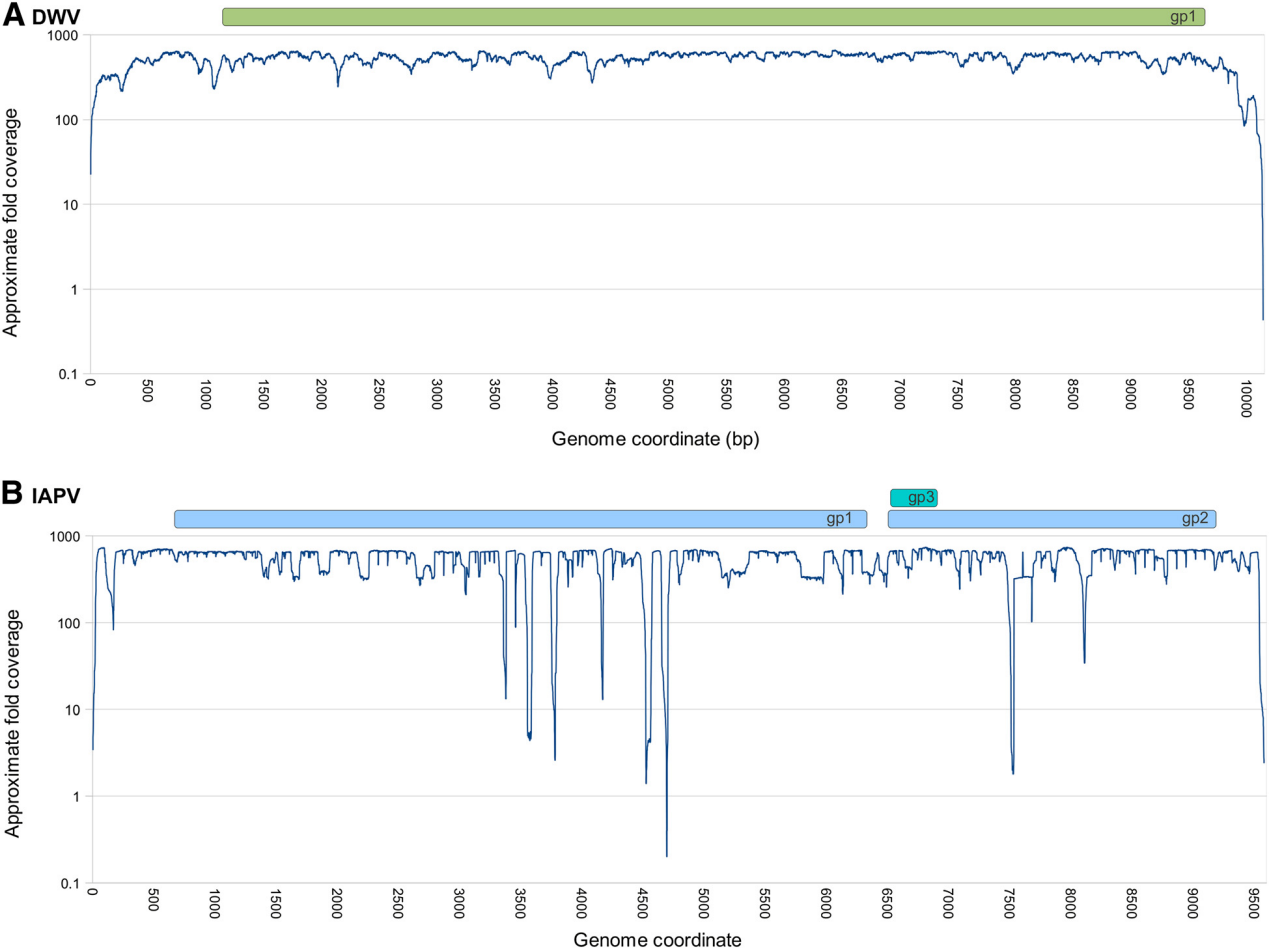
**Read depth along the length of each viral genome.A**. Read depth for DWV. **B**. Read depth for IAPV. Coding regions are represented at the top of each panel in this and all subsequent figures depicting sliding-window analyses. The maximum coverage is constrained by default limits used in the analysis pipeline. The sharp minima within IAPV were generally found in multiple samples and were virtually unchanged when mapping criteria were relaxed from 4% to 8% divergence, suggesting these gaps are caused by an unknown bias during the generation of sequencing libraries rather than sequence divergence from the reference.

Figure 1 shows the distribution of read coverage for each genome, averaged across all samples in which the virus was detected. The maximum coverages are driven by our use of coverage cutoffs in samtools [31] to generate alignment files. The coverage predictably declines at both the 3’ and 5’ ends of each genome due to the difficulty of getting complete cDNA coverage, but is otherwise uniform across the DWV genome. In contrast, there are several narrow windows of unexplained low coverage in IAPV. One possibility is that the sequenced strains are divergent in those regions and fail to map under the algorithm parameters used. However, adjusting the bwa [23] parameters to a more permissive mismatch level (8% rather than 4% per read, with 2 mismatches in the 28-bp seed region) had no tangible effect on the pattern of read mapping (results not shown). Coverage gaps were consistent across multiple samples, indicating a biological rather than stochastic cause, e.g. a compositional or secondary-structure barrier to cDNA synthesis. A perhaps related observation was an extremely high variance in coverage in the BRL and WEAV samples for IAPV only (data not shown), as well as a high number of indels observed when reads were mapped with stampy [32], an indel-sensitive alignment program. Thus, there appear to be technical issues associated with shotgun sequencing of IAPV that could complicate future reseqeuncing efforts, although the affected regions were short and we do not believe the coverage gaps qualitatively affect our results.

### Phylogeny and diversity of viral samples from consensus sequences

**Figure 2:**
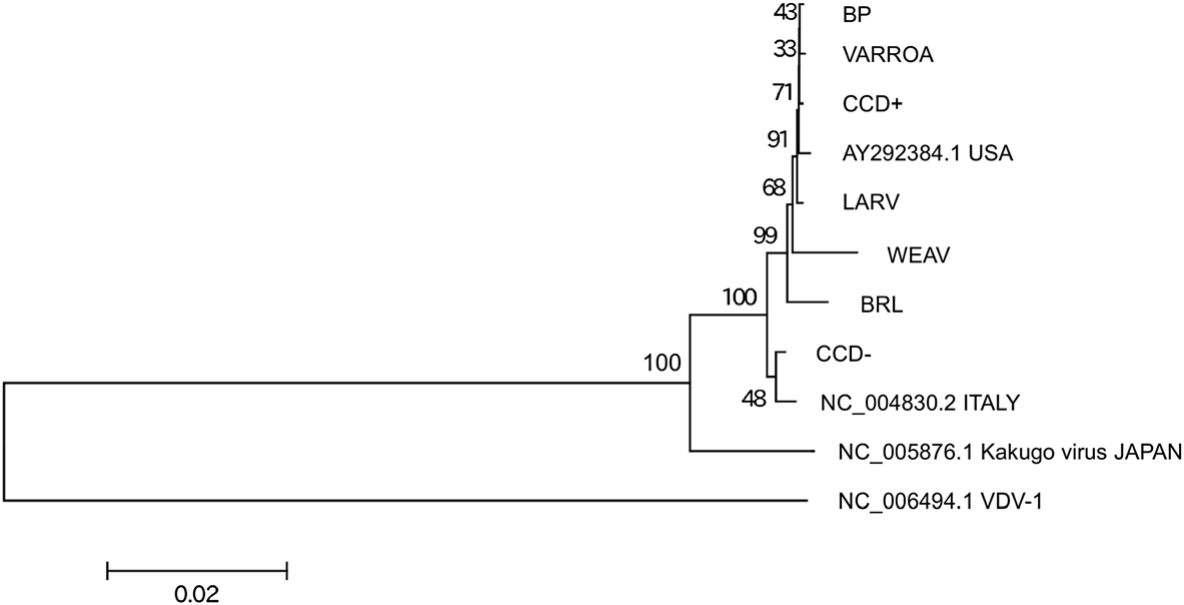
**Phylogeny of DWV based on GenBank accessions and deep sequencing reads.** Deep sequencing samples and consensus-sequence generation are described in the text. The tree was constructed with MEGA5 [26] using neighbor-joining and nucleotide genetic distances estimated with the Tamura 3-parameter model (T93). A gamma distribution of rates was estimated with parameter value 5.0. Bootstrap support for each node is indicated based on 1,000 resampled replicates. GenBank accession numbers are given where applicable, and the country of origin for each DWV accession is indicated (Kakugo virus is a DWV accession isolated from Japan).

Consensus sequences of DWV and IAPV were generated for each sample in order to estimate their phylogenetic relationship with each other and with additional accessions obtained from GenBank. Neighbor-joining, minimum evolution, and maximum parsimony methods produced broadly concordant tree topologies, as did different models of nucleotide mutation (we show neighbor-joining trees based on pairwise distances calculated with the Tamura 3-parameter model and a gamma distribution coefficient of 5.0 [26]). Most DWV sequences from the U.S. clustered apart from the reference genome (accession NC_004830.2), which was isolated in Italy, at 100% bootstrap support (Figure 2). The CCD- sample was the exception, but this sample also had the lowest coverage and a number of gaps where no consensus could be inferred. Most U.S. sequences are very similar, as indicated by their short branch lengths, including the consensus sequence derived from the VARROA sample.

**Figure 3:**
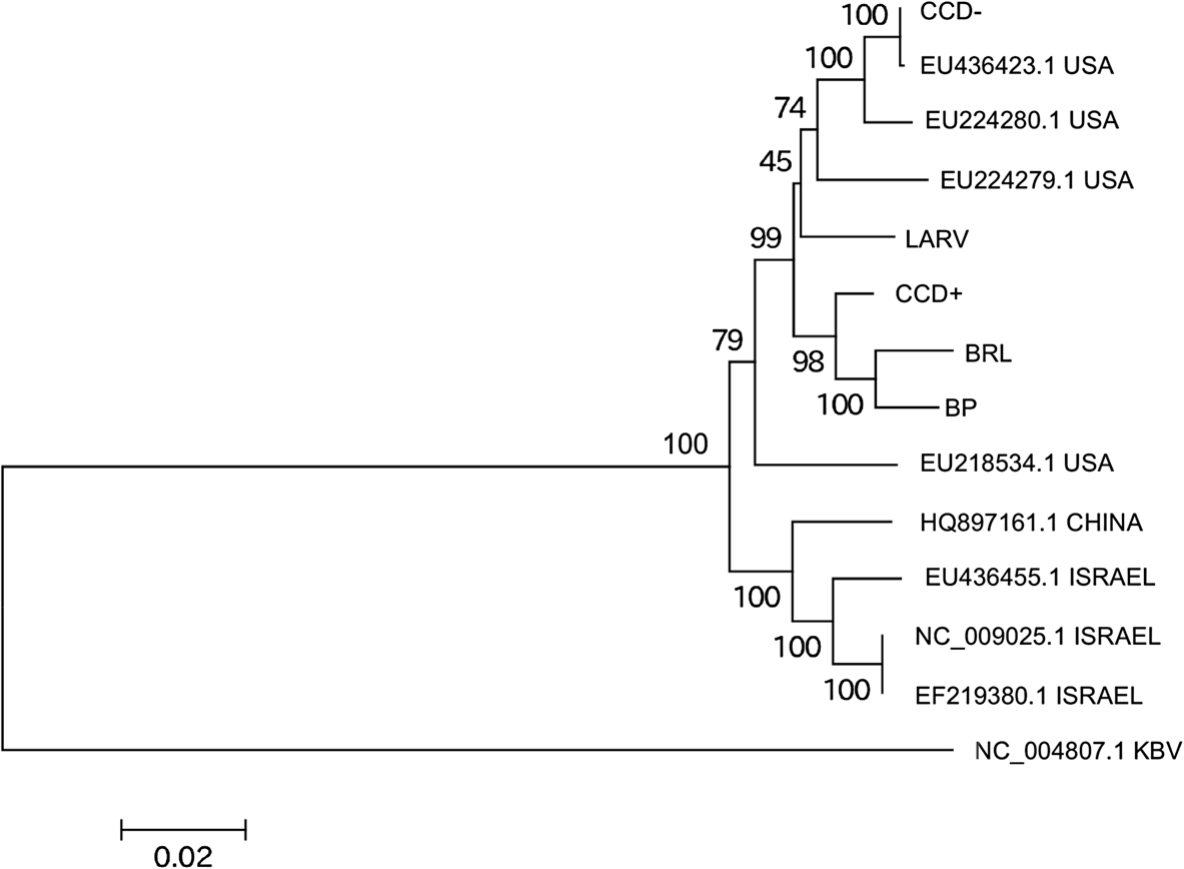
**Phylogeny of IAPV based on GenBank accessions and deep sequencing reads.** Deep sequencing samples and consensus-sequence generation are described in the text. The tree was constructed with MEGA5 [26] using neighbor-joining and nucleotide genetic distances estimated with the Tamura 3-parameter model (T93). A gamma distribution of rates was estimated with parameter value 5.0. Note difference in genetic-distance scale compared with Figure 2. Bootstrap support for each node is indicated based on 1,000 resampled replicates. GenBank accession numbers are given where applicable, and the country of origin for each IAPV accession is indicated.

Compared with DWV, the branch lengths of the IAPV phylogeny (Figure 3) are substantially longer (note difference in scale bar between Figure 2 and Figure 3). There is also bootstrap support for distinct clades within IAPV, in contrast to DWV. Palacios et al. [21] had previously identified comparable genetic structure in IAPV; specifically, they inferred three distinct lineages and found that these subdivisions were robust when different genomic regions were examined separately. Even so, all North American sequences cluster together at 100% bootstrap support relative to accessions from other continents. This was true regardless of whether accession NC_009025.1, an Israeli isolate [18] present in the NCBI Genomes database, or U.S. accession EU224280.1 was used as the mapping reference to generate consensus sequences.

We next examined SNP variation among sample consensus sequences and GenBank whole-genome accessions, to assess how these patterns diverge from their expected values under neutral evolution. In the absence of recombination, single-molecule viral genomes are expected to behave as one locus with respect to natural selection, showing only stochastic variation among windows. However, recombination has been reported for both of these viruses [21,29], so we first investigated whether the set of consensus sequences showed evidence of recombination, using Hudson’s test as implemented in DnaSP [25]. Hudson’s test indicated very low rates of recombination for DWV (R parameter = 0.001 per genome) but measureable rates of recombination for IAPV (R = 79.7 per genome, or 0.0085 between adjacent sites). These tests and the results to follow imply that variation in selective pressures among genomic regions can indeed contribute to local deviations in population-genetic parameters. In fact, we suspect the lack of evidence for recombination in DWV in these samples may be due to the low overall diversity rather than a genuine lack of recombination, given that recombinants between DWV and the related virus VDV-1 have been detected [29].

**Figure 4:**
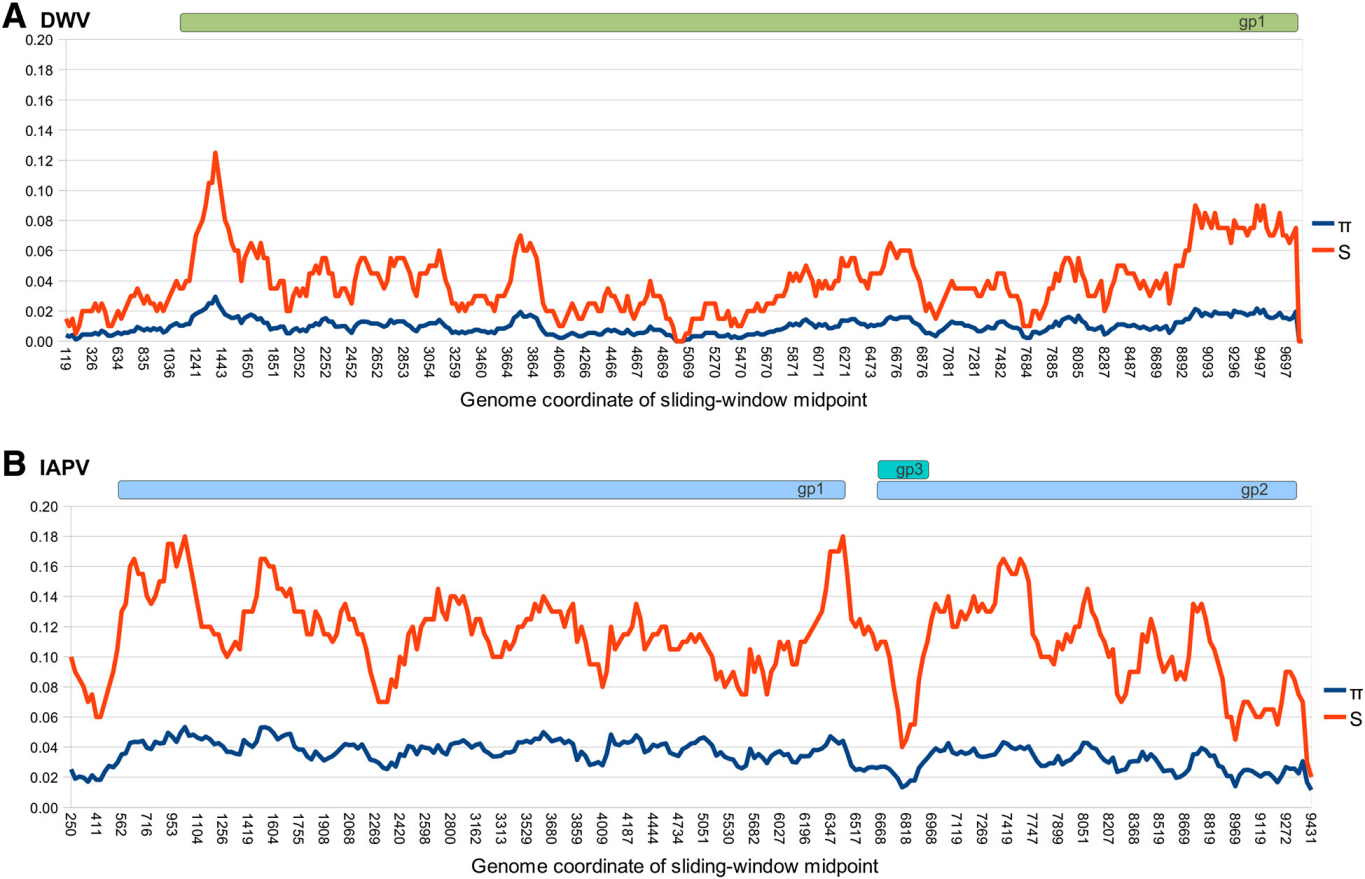
**Sliding-window analysis of polymorphism among consensus sequences for each virus.A**. DWV among-sample polymorphism. **B**. IAPV among-sample polymorphism. Nucleotide diversity (π) and the number (presented as a proportion) of segregating sites (S) are plotted for windows of 200 bases with a step-size of 25, and were calculated with DnaSP [25]. Gaps introduced by including GenBank accessions of different lengths are ignored in the calculations, resulting in a different alignment length and relative coding positions compared with Figure 1.

Figure 4 shows sliding-window analyses of π and S for each virus. S is shown as a proportion of polymorphic sites in the window, not the absolute number. The genome average π was six times greater for IAPV than DWV (0.062 and 0.010, respectively). IAPV has a distinct local minimum of π associated with the small peptide gp3, which overlaps the 5’ end of gp2 in the +2 frame. This minimum reflects the greater nucleotide conservation inherent to overlapping reading frames, as all sites are first- or second-position sites for one or the other peptide. Both viruses show drops in diversity at the extreme 3’ (noncoding) end of the genome.

**Figure 5:**
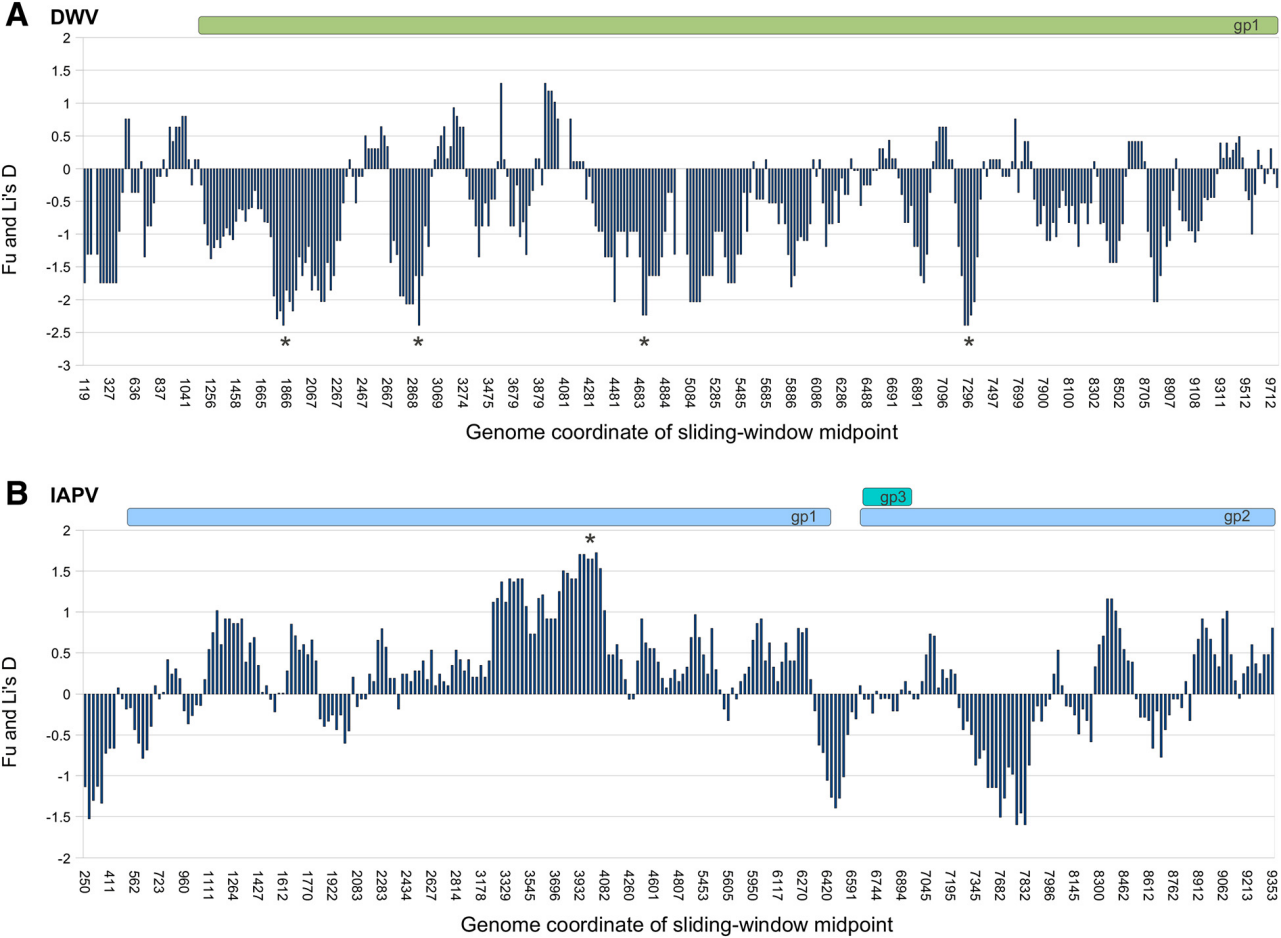
**Sliding-window analysis of Fu and Li’s D for each virus.A**. Values of D for DWV. **B**. Values of D for IAPV. Values were calculated with DnaSP [25] using 200-base windows and a step-size of 25 base. Asterisks indicate windows with statistically significant deviations from neutral expectation at P < 0.05. The outgroup for this coalescent-based statistic is VDV-1 for DWV and KBV for IAPV; gaps introduced by alignment to these outgroups are ignored, resulting in a different alignment length and relative coding positions compared with Figure 1.

Figure 5 shows sliding-window values of Fu and Li’s D statistic [25,33], which tests for significant departures from neutral evolution under a coalescent model and phylogenetic outgroups. The test considers whether rare or high-frequency alleles are over-represented within a set of polymorphic sites. Significant deviations of this statistic from zero indicate a violation of one or both of the two principle assumptions of the method, absence of selection and constant population size. Since the D statistics of freely recombining loci are expected to be affected similarly by demography but differently by selection, strong outlier values indicate distinct patterns of natural selection acting in those regions. DWV has several windows with significantly negative D statistics, marked by asterisks in Figure 5A, suggesting purifying selection is acting most strongly at these sites, whereas no windows were significantly negative for IAPV. The overall D of the DWV was also much lower than for IAPV (−1.773 and −0.431, respectively), which is consistent with an hypothesized population bottleneck that would likely have occurred if only a restricted set of ancestral DWV genotypes were vectored by the Varroa mite during its recent, global spread [22]. Some regions with low D values are known to be functionally constrained, such as the internal ribosomal entry site (IRES) upstream of the IAPV gp2 polyprotein, which forms a conserved secondary structure [34] that interacts with the ribosome. However, other sites that we might have expected to show low D values did not, such as the IRES of DWV, which has been mapped to the 300 bp 5’ of the polyprotein coding sequence [35]. Interestingly, a significantly high D value in Figure 5B suggests that selection has acted to maintain high-frequency variants in the protease region of IAPV gp1. Of course, selection acting on elements that are multi-partite or small relative to the size of the sliding window may not be evident. For instance, we identified a 40-bp region in the 3' noncoding region of DWV (Additional file 1: Figure S1) that is invariant among GenBank accessions, consensus sequences, and the related virus VDV-1, and has very low segregating variation within samples as well. Yet this area of strong sequence conservation is not evident in the sliding-window analysis.

### Within-sample diversity

The results of the previous section were based on the consensus base at each position in each sample, and did not consider variation segregating within a sample. Within-sample patterns of polymorphism give further power for identifying patterns of natural selection. In this section, we calculate haplotype diversity at SNPs within each sample. Haplotype diversity is numerically equivalent to expected heterozygosity in the case of diploid organisms, and is used here instead of π because the latter cannot be readily calculated from short-read data because the linkage of polymorphisms is generally unknown. However, an ascertainment bias is introduced to SNP detection by variation in the depth of sequence coverage within each sample, because the chance of identifying an alternative allele, i.e. classifying a genomic position as a SNP, increases with the number of reads mapped to that position. We therefore calculated haplotype diversity for sliding-window analysis using only sites with a minimum coverage of ten reads. To compare the genome-mean values between the two viral species, we further restricted sites to those that also had haplotype diversity > 0.05, in order to minimize the contribution of low-frequency alleles.

**Figure 6:**
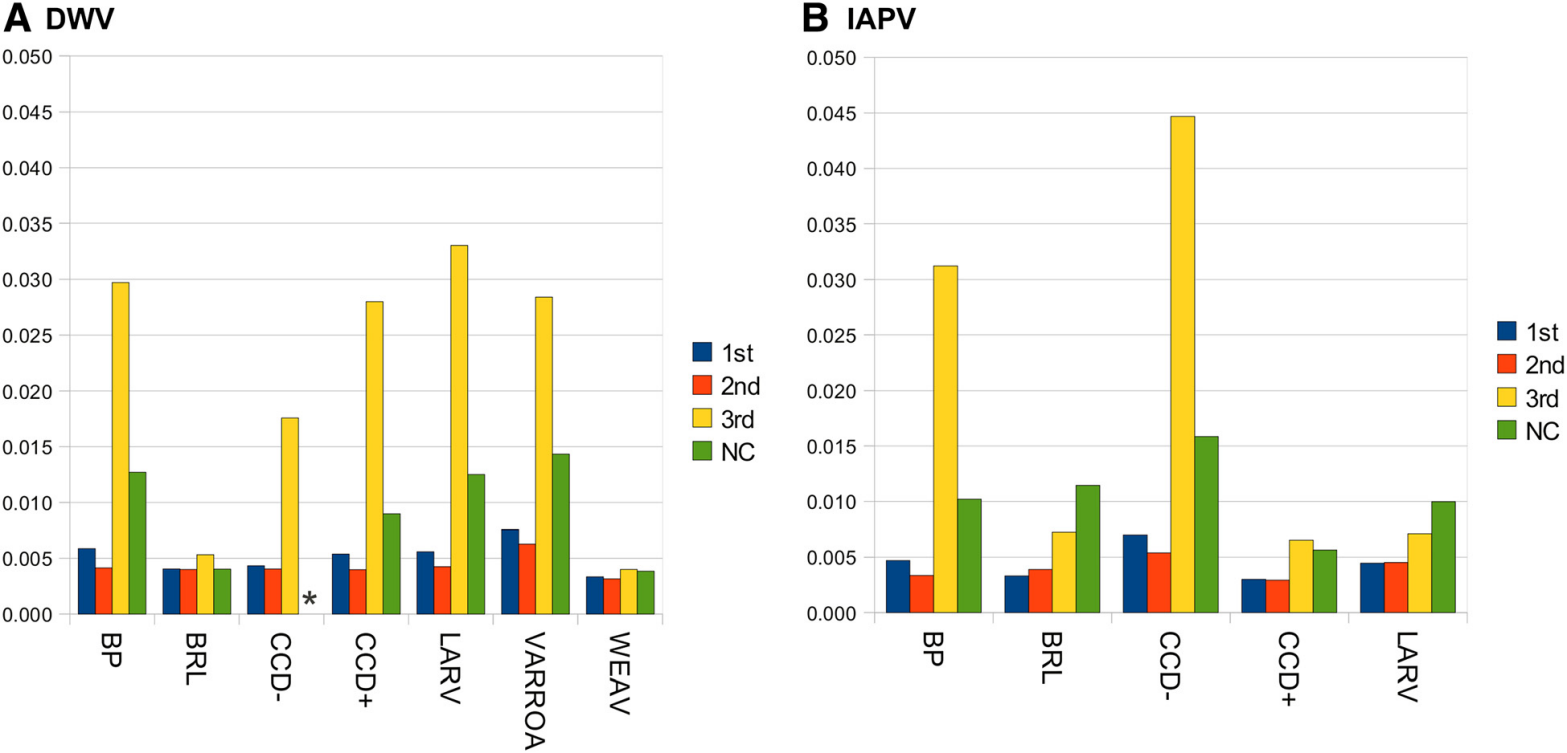
**Mean haplotype diversity at SNPs by codon position for each sample.** Within each sample, only sites with read-coverage of ten or more were considered. **A**. DWV haplotype diversity. The asterisk indicates that no noncoding sites were polymorphic for the CCD- sample. **B**. IAPV haplotype diversity.

**Figure 7:**
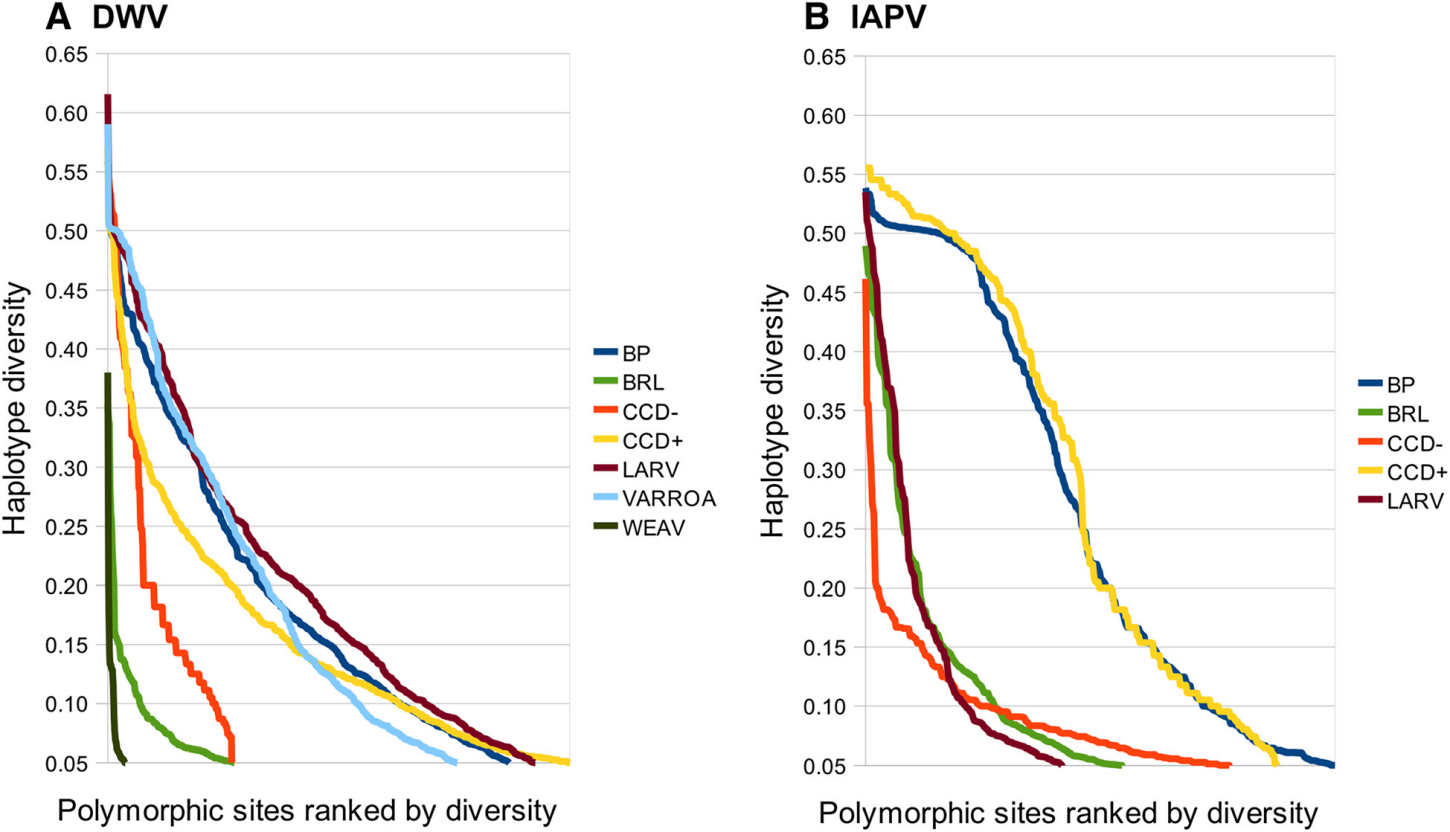
**Haplotype-diversity at SNPs in each sample for each virus, ranked in descending order for first and third codon positions.A**. DWV ranked diversity values. **B**. IAPV ranked diversity values. The spectrum of polymorphism levels is distinctly lower for some samples, suggesting different population-genetic histories. A 10-read threshold was required to calculate haplotype diversity at a site.

Figure 6 shows the sample mean SNP diversity for each virus and each sample, calculated separately for first, second, and third codon positions and for noncoding positions (the positions coding for gp3 of IAPV were excluded because they code in two frames). Although DWV has lower among-population variation, the level of variation segregating within populations is comparable to IAPV. For both viruses, the mean third-position diversity varied greatly among populations. Since third position substitutions are generally the most weakly selected, variation at these positions may be more indicative of demographic factors such as the number of hosts or time since initial infection. The number of high-diversity sites also varies among samples. Figure 7 shows the diversity estimate at each SNP in each sample, ranked by descending value and separated into first and third codon positions, a convenient representation comparable to the site frequency spectrum. For both viruses, the proportion of high-diversity SNPs is distinctly lower in three samples compared with the remainder, two of which are the same for each virus (BRL and CCD-). This difference is particularly intriguing for the CCD- sample, as the total number of sampled bees (Table 1) was slightly greater than for CCD+ and the geographic areas of sampling were broadly overlapping [4]. Why some samples accrue less variation than others could be related to the total number of infected individuals included or some other demographic factor, and merits further investigation.

**Figure 8:**
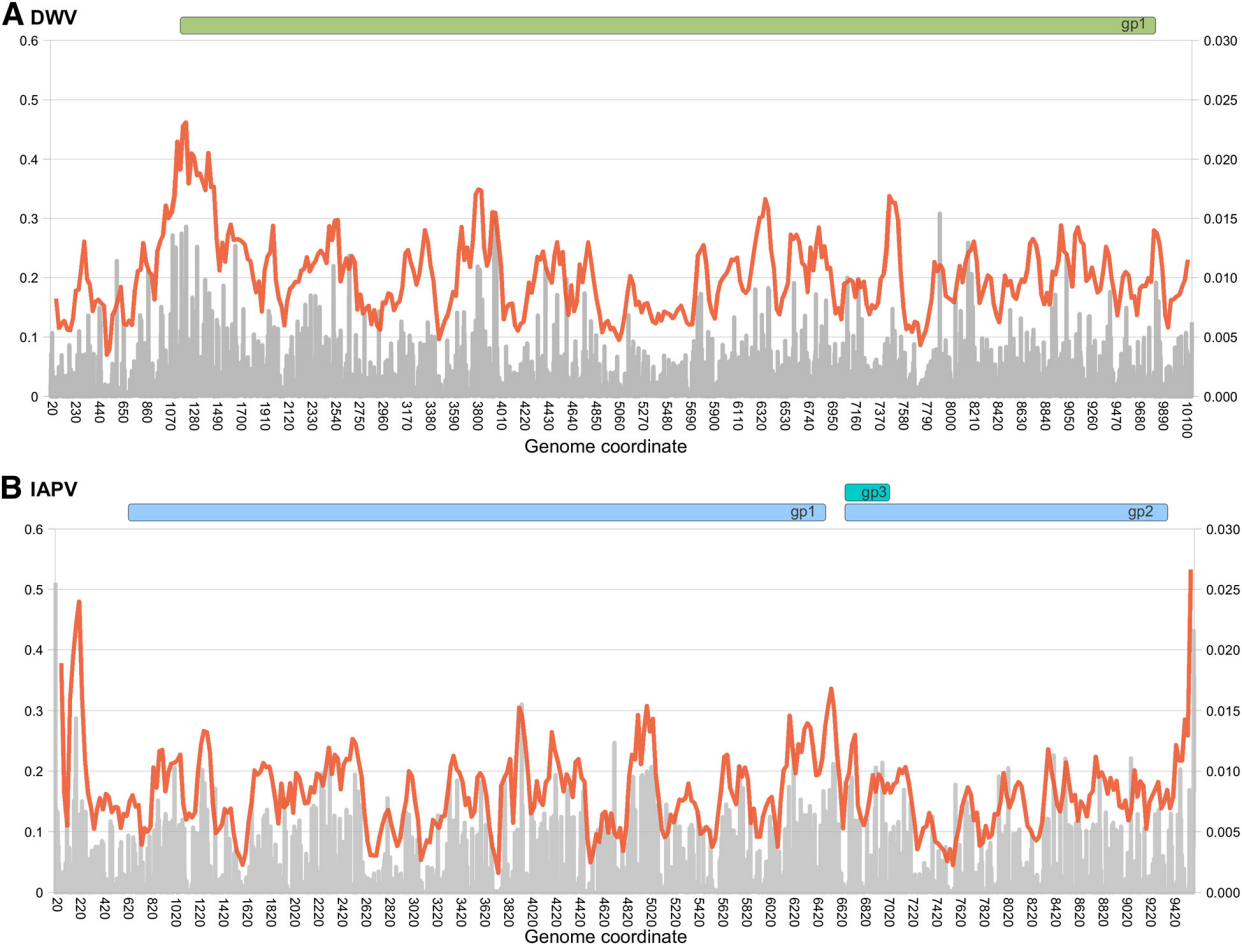
**Genomic distribution of haplotype diversity at SNPs within each virus.A**. DWV haplotype diversity. **B**. IAPV haplotype diversity. Grey bars represent values at each site averaged across all samples with at least 10× coverage at that site (left axis). The orange line is the mean of a sliding window of 100 bases with a step size of 25 bases (right axis).

Figure 8 shows per-site levels (grey bars and left axis) and sliding-window averages (orange line and right axis) of mean haplotype diversity, which presumably reflect genomic patterns of mutation-selection balance. The highest diversities in DWV were observed around the initiation of translation, whereas IAPV had high values at the 5’ and 3’ extremes of the genome.

### Genome scans of F_ST_

**Figure 9:**
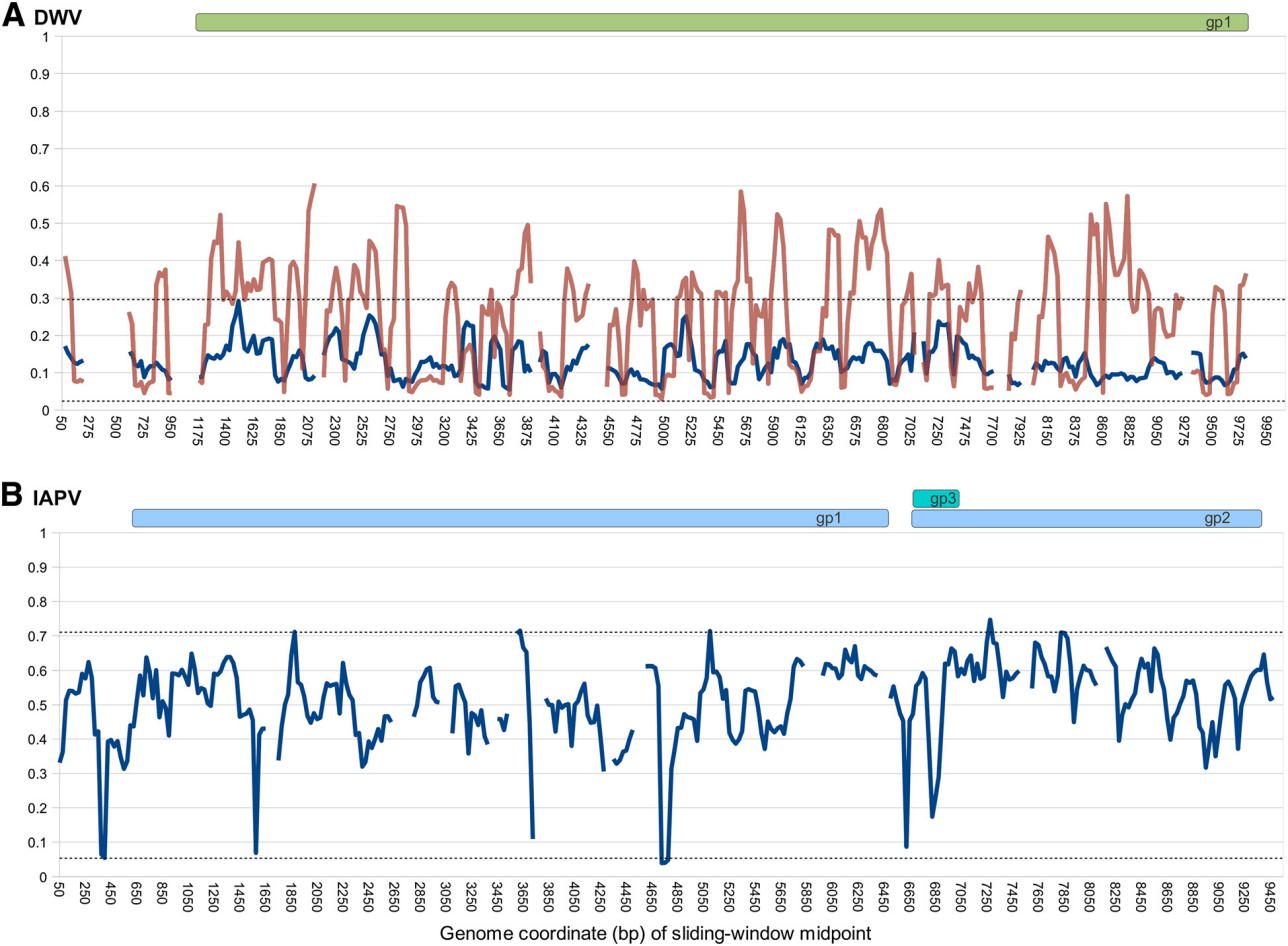
**Sliding-window analysis of F**_**ST**_**at SNPs among viral samples.A**. DWV values of F_ST_. **B**. IAPV values of F_ST_. Values were calculated with popoolation2 [28] using a window size of 100 bases and a step size of 25 bases. A minimum read count of 4 per site at a minimum of 40% of sites was required within each window or else no estimate was calculated (shown as gaps in the continuity of the line).

While Figure 8 can provide some hints as to viral regions that are more or less constrained based on mean polymorphism levels, genome scans of F_ST_ provide stronger evidence of sites that are either more conserved or more divergent than the genome average. F_ST_ is a sensitive index of allele-frequency divergence that is scaled from zero to one (see [36] for a review); because of this normalization, species with low overall polymorphism can nonetheless have high F_ST_ among populations (indicating low gene flow or diversifying selection), or vice versa. One complication, however, is that the minimum number of reads per site must be met for all samples analyzed, such that the number of shared SNPs declines rapidly as the minimum coverage requirement increases. We therefore used a fairly lenient coverage requirement for our sliding-window estimates: at least 4 reads per site and at least 40% of the sites in the window meeting this criterion. Increasing the coverage requirement did not change the qualitative pattern for either genome (data not shown), but greatly increased the number of windows for which no value could be calculated (seen as gaps in Figure 9). To evaluate the significance of local variation in F_ST_, we randomized genomic positions to generate 100,000 100-bp windows for each virus. Values less than the 0.05 percentile or greater than the 99.95 percentile are below or above the dotted lines in Figure 9, respectively.

The two viruses show strikingly different patterns of F_ST_ across their genomes. DWV oscillates frequently between a higher and a lower band of values, such that the histogram (Additional file 2: Figure S2) is strongly bimodal, with one mean around 0.07 and another mean around 0.32. This pattern appears to be driven by genetic drift in the BRL and WEAV samples, which have the longest branch lengths in Figure 1, low overall diversity compared with the other DWV samples (Figure 7), and pairwise per-window F_ST_ values that are much higher on average than among the remaining samples. Excluding these two samples, the mean F_ST_ values among the remaining DWV samples are much lower and less variable (blue line in Figure 9A) than when all seven populations are included (orange line). While diversifying selection is another cause of high F_ST_, the large number of sites with high F_ST_ and the similarity of the values indicates that genetic drift, perhaps due to a founder effect, is the more parsimonious interpretation. It is nonetheless surprising to see such rapid swings in local F_ST_, and it suggests that strongly constrained sites occur in numerous small clusters throughout the genome. The dotted lines in Figure 9A represent the upper and lower 0.5% of the distribution of randomized FST values, calculated after excluding the BRL and WEAV samples. The sliding-window F_ST_ estimates for the remaining five samples (blue line) fall within the extremes of this distribution, suggesting predominantly stochastic variation. In contrast to DWV, IAPV has a unimodal distribution of FST values across the genome and a higher mean value of approximately 0.48 (Figure 9B and Additional file 2: Figure S2), although several strong outliers of low FST are apparent. This pattern suggests that there are only a few windows within which purifying selection is strong and consistent across samples, resulting in local haplotype frequencies that are far more uniform than the genome average.

## Conclusions

In this study, we have performed an initial, random survey of genomic variation in two honey bee viruses, DWV and IAPV, of established relevance to honey bee health. Even though the sequencing was originally undertaken for diverse, unrelated reasons, our results demonstrate the utility of population-genomic data for detecting important aspects of viral biology. More directed sequencing efforts using virus-enriched samples and controlled study designs should provide even greater power to discern evolutionary forces acting on genomic variation.

The data presented provide support for the idea that the spread of the Varroa mite has enforced a selective sweep on DWV sequences, by giving high fitness to genotypes that can replicate in and be transferred by the parasite. DWV replication is known to occur in Varroa mites [15-17] and the introduction of Varroa to Hawaii was associated with both a great increase in DWV infections and a great decrease in DWV diversity in that state [22]. The latter data strongly implicate selection for vectoring by Varroa as a driver of genetic uniformity in DWV. This hypothesized selective sweep is consistent with the lower diversity and divergence found among populations of DWV relative to IAPV, despite comparable levels of within-population variation. While there is correlational evidence that IAPV can be transmitted by Varroa mites [37], the lower frequency of IAPV relative to DWV and greater genetic distances among IAPV samples suggest that Varroa is not a frequent vector of IAPV in natural populations.

The high read count of DWV in the VARROA sample (Table 1) is consistent with replication in the Varroa mite, although the various methods used to generate these data preclude a reliable reference gene for relative quantification across samples or even determining whether the virus was present within tissues other than the gastrointestinal tract. Interestingly, the consensus sequence and polymorphism levels of this sample were very similar to honey-bee derived samples, implying that no large-scale genotypic shift occurs during replication in that host. More definitive evidence that isolates from either host have equivalent fitness in the other is needed, however. In the absence of a culture method for DWV, a useful approach would be to analyze viral growth rates and sequence divergence among replicated infections in each host, initiated from natural inoculates from the same or alternate host. Such data would demonstrate a replicable effect of host source on the fitness of the virus. A similar approach could be taken using inoculates from the few remaining areas of honey-bee cultivation that are free of the Varroa mite. It would also be of interest to investigate DWV diversity in Africanized populations, which experience much less parasitism by Varroa. As some honey bee RNA viruses have been detected by PCR in other sympatric pollinators [38], indicating the possibility of alternate insect hosts, deep sequencing can also shed light on the process of adaptation to alternative hosts.

In macroscopic eukaryotes that do not disperse widely, high F_ST_ at neutral loci is expected to arise from a lack of gene flow between separate interbreeding populations. This interpretation is dubious for RNA viruses, however, which can rapidly reach selection-mutation equilibrium even after single-genome bottlenecks. Thus, high or low F_ST_ values more likely indicate selective pressures rather than dispersal rates of virions. Multiple narrow windows of low F_ST_ values were strikingly evident among the IAPV samples, for example, suggesting strong and concordant selective pressures shaping polymorphism levels at those sites. Even so, the genome-wide pattern of higher F_ST_ between DWV sequences from the WEAV and BRL samples relative to other samples suggests that founder effects do occur and can substantially impact the spectrum of genotypes observed. In fact, phylogenetic analysis of both species shows geographic structuring of variation at large scales, as U.S. samples tend to cluster together relative to samples from other continents. This continental-scale genetic structure has apparently persisted despite historical trade in bees and/or bee products that likely moved viral particles between continents. IAPV exhibits phylogenetic structure within the U.S. as well, but it is not yet known whether there is also a spatial component of this variation.

Both genomes had low among-sample diversity at the 3’ end of the alignments (Figure 4), and an example of strong conservation in an approximately forty-base window was shown in Additional file 1: Figure S1. The 3’noncoding region of the picornavirus genome is believed to be a major *cis*-acting regulator of negative-strand RNA synthesis by promoting recognition of the replication complex [39,40]. An important role in genome replication could therefore underlie the low level of nucleotide variation in this region, but infectious clones and cell-culture propagation methods are presently unavailable to support reverse genetic studies of DWV. Interestingly, the highest haplotype diversities at individual IAPV SNPs occurred at the extreme 5’ and 3’ sites (Figure 8); the latter do not influence Figure 4 because of alignment gaps for some IAPV accessions and consensus sequences. The greater diversity at these sites could potentially indicate higher rates of mutation associated with the replication of genome ends.

A distinctive feature of DWV is a peak of sequence diversity at the 5’ end of the coding sequence that was observed both among populations (π and S) and within populations (haplotype diversity). In fact, Fu and Li’s D was weakly positive upstream of the coding region rather than negative, contrary to what might be expected based on its putative role in binding the host ribosome [35]. It is not clear whether windows that did have significantly negative values of D are experiencing stronger purifying selection than other regions or are simply the extremes of a distribution that has been shifted into predominantly negative values. It should also be noted that this statistic was applied to consensus sequences rather than haplotypes; the latter would likely provide greater resolution of the extent of among-region variation in selective pressures. However, our results help define interesting regions to target for amplicon-based deep sequencing with longer or paired-end reads, so that haplotype structure can be retained.

## Competing interests

The authors declare they have no competing interests.

## Authors’ contributions

RSC conceived the study, performed population genomic analyses, and drafted the manuscript. HB and BD participated in the design of the analysis and interpretation of the results. HB, DV, DW, YC, and JE collected samples and contributed sequencing data. All authors read and approved the final manuscript.

## Supplementary Material

Additional file 1: Figure S1 A. An alignment of a portion of the 3’ untranslated region of DWV, showing conservation among all consensus sequences and accessions, including VDV-1. The CCD- and CCD+ samples are not shown due to insufficient read coverage in this region. B. Mean haplotype diversity (10-read minimum coverage) in the 3’ untranslated region of DWV, showing a lack of within-population variation in the same window (shaded in blue) that is marked in panel A. Click here for file

Additional file 2: Figure S2 Histograms of F_ST_ values from the sliding-window analysis in Figure 9. For DWV, the values are for all seven positive samples including the outlier samples BRL and WEAV. Click here for file
