# Supplementary figures and images for: Population-genomic variation within RNA viruses of the Western honey bee, Apis mellifera, inferred from deep sequencing
Source: BMC Genomics. 2013 Mar 7;14:154. doi: 10.1186/1471-2164-14-154 (PMC3599929; doi:10.1186/1471-2164-14-154)

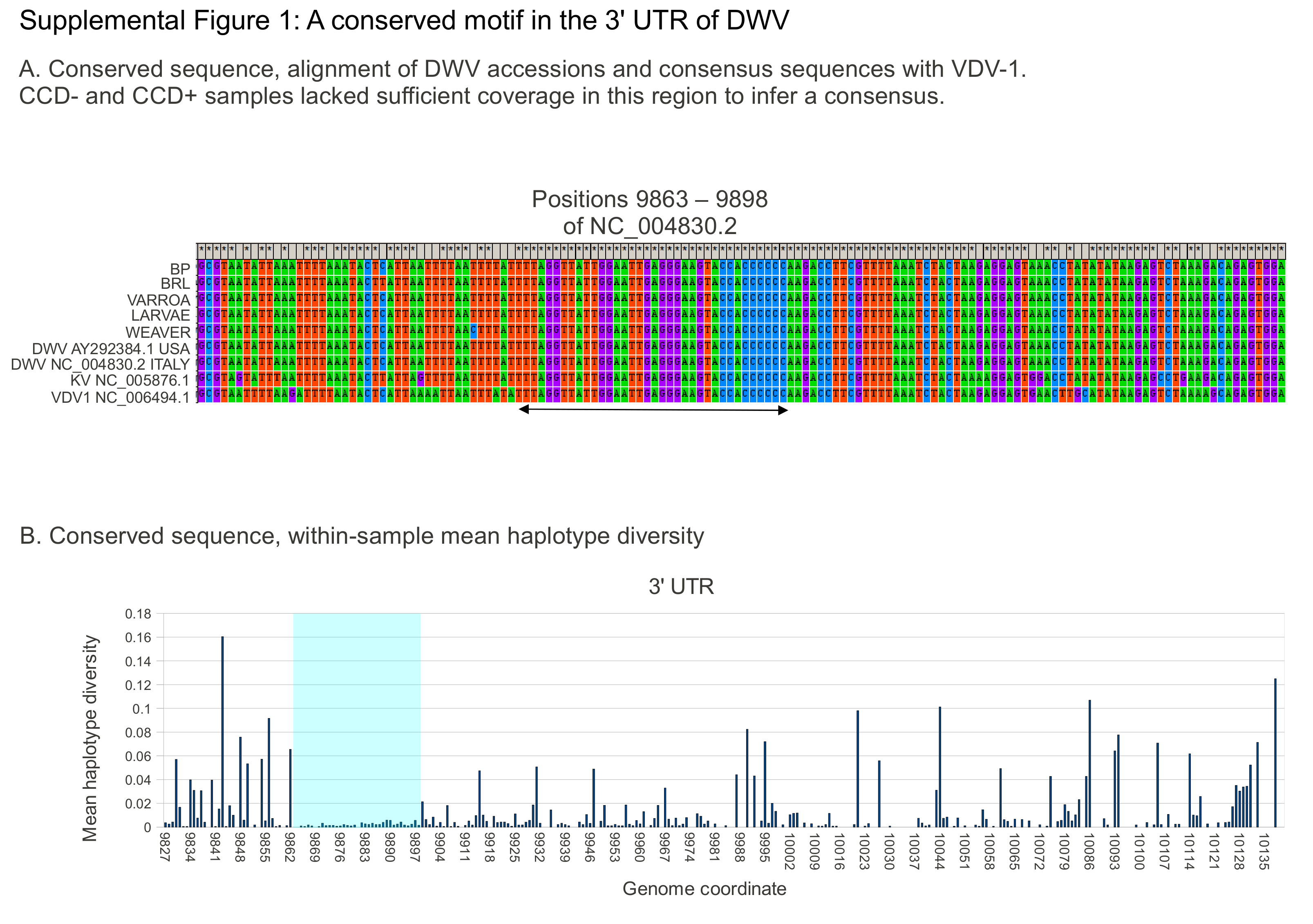

Supplement: Additional file 1: Figure S1 — A. An alignment of a portion of the 3’ untranslated region of DWV, showing conservation among all consensus sequences and accessions, including VDV-1. The CCD- and CCD+ samples are not shown due to insufficient read coverage in this region. B. Mean haplotype diversity (10-read minimum coverage) in the 3’ untranslated region of DWV, showing a lack of within-population variation in the same window (shaded in blue) that is marked in panel A. [file 1471-2164-14-154-S1.tiff]

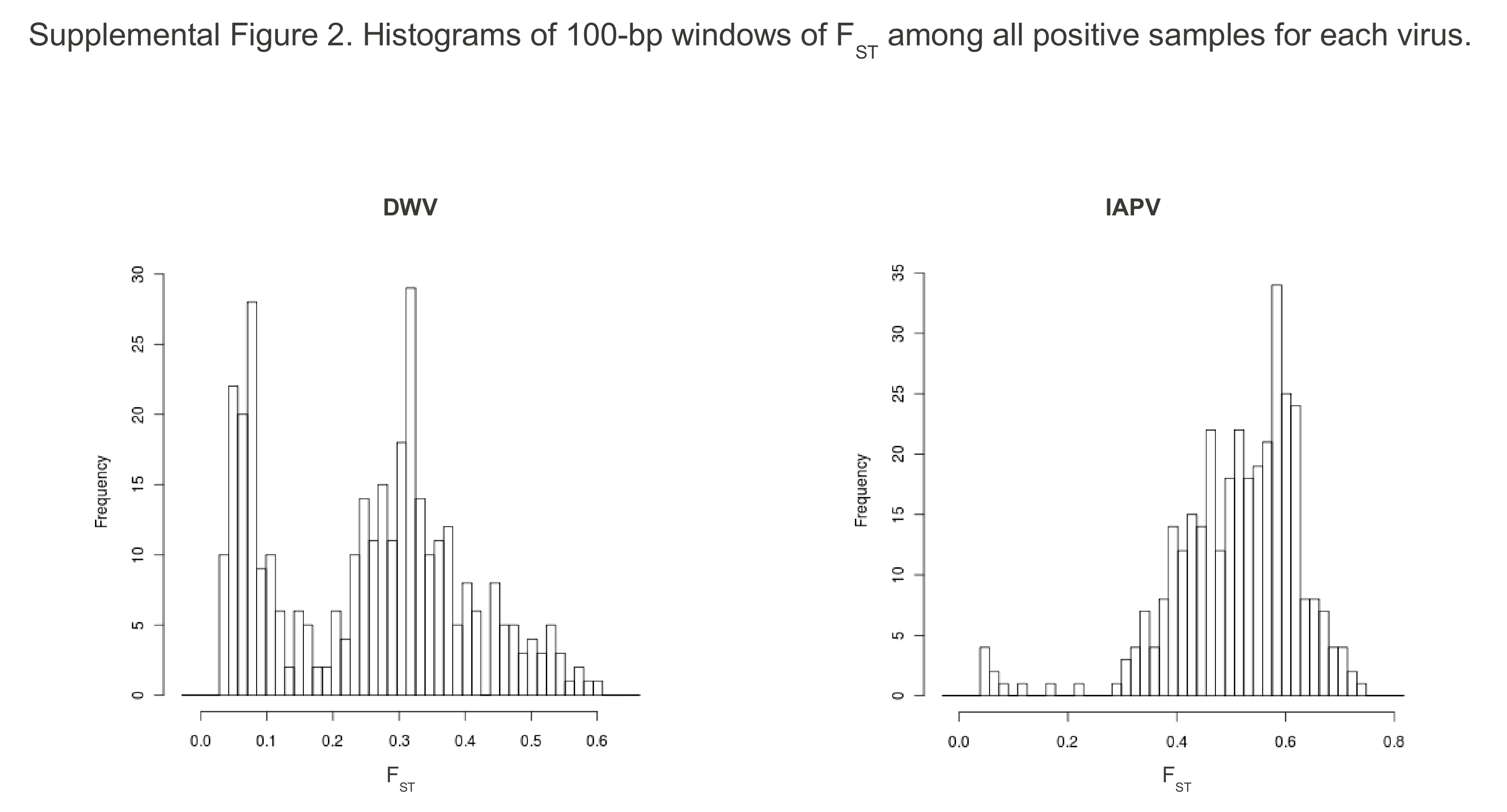

Supplement: Additional file 2: Figure S2 — Histograms of FST values from the sliding-window analysis in Figure 9. For DWV, the values are for all seven positive samples including the outlier samples BRL and WEAV. [file 1471-2164-14-154-S2.tiff]
